# Effect of the Planet Health Intervention on Eating Disorder Symptoms in Massachusetts Middle Schools, 2005–2008

**DOI:** 10.5888/pcd9.120111

**Published:** 2012-11-29

**Authors:** S. Bryn Austin, Jennifer L. Spadano-Gasbarro, Mary L. Greaney, Emily A. Blood, Anne T. Hunt, Tracy K. Richmond, Monica L. Wang, Solomon Mezgebu, Stavroula K. Osganian, Karen E. Peterson

**Affiliations:** Author Affiliations: Jennifer L. Spadano-Gasbarro, Mary L. Greaney, Monica L. Wang, Karen E. Peterson, Harvard School of Public Health, Boston, Massachusetts; Emily A. Blood, Tracy K. Richmond, Stavroula K. Osganian, Boston Children’s Hospital, Boston, Massachusetts; Anne T. Hunt, Hunt Consulting Associates, Lyme, New Hampshire; Solomon Mezgebu, Massachusetts Department of Public Health, Boston, Massachusetts.

## Abstract

**Introduction:**

The Planet Health obesity prevention curriculum has prevented purging and abuse of diet pills (disordered weight control behavior [DWCB]) in middle-school girls in randomized trials, but the effects of Planet Health on DWCB when implemented by schools under dissemination conditions are not known.

**Methods:**

Massachusetts Department of Public Health and Blue Cross Blue Shield of Massachusetts disseminated Planet Health as part of the 3-year, Healthy Choices obesity prevention program in middle schools. We conducted an evaluation in 45 schools from fall 2005 to spring 2008. We gathered data from school staff to quantify intervention activities, and we gathered anonymous cross-sectional survey data from students on DWCB at baseline and Year 3 follow-up (n = 16,369). Multivariate logistic analyses with generalized estimating equations examined the effect of intervention activities on odds of students reporting DWCB at follow-up.

**Results:**

Students in schools reaching a high number of youth with Planet Health lessons on reducing television viewing had lower odds of DWCB at follow-up (odds ratio [OR], 0.80 per 100 lesson-exposures; 95% confidence interval [CI], 0.74–0.85). In addition, reduced odds of DWCB at follow-up were found in schools with active staff teamwork (OR, 0.76; 95% CI, 0.66–0.86) and the presence of programs addressing television viewing goals with staff (OR, 0.38; 95% CI, 0.28–0.53).

**Conclusion:**

Combined evidence from efficacy and effectiveness trials and now from dissemination research indicates that appropriately designed obesity prevention programs can achieve DWCB prevention on a large scale.

## MEDSCAPE CME

Medscape, LLC is pleased to provide online continuing medical education (CME) for this journal article, allowing clinicians the opportunity to earn CME credit.

This activity has been planned and implemented in accordance with the Essential Areas and policies of the Accreditation Council for Continuing Medical Education through the joint sponsorship of Medscape, LLC and Preventing Chronic Disease. Medscape, LLC is accredited by the ACCME to provide continuing medical education for physicians. 

Medscape, LLC designates this Journal-based CME activity for a maximum of 1 **AMA PRA Category 1 Credit(s)™**. Physicians should claim only the credit commensurate with the extent of their participation in the activity.

All other clinicians completing this activity will be issued a certificate of participation. To participate in this journal CME activity: (1) review the learning objectives and author disclosures; (2) study the education content; (3) take the post-test with a 70% minimum passing score and complete the evaluation at www.medscape.org/journal/pcd (4) view/print certificate.

**Release date: November 28, 2012; Expiration date: November 28, 2013**

### Learning Objectives

Upon completion of this activity, participants will be able to:

- Analyze the Planet Health program for school-aged children
- Distinguish the impact of the Planet Health intervention on the prevalence of DWCB in the current study
- Analyze variables associated with significant reductions in DWCB in the current study

**CME EDITOR**

Caran Wilbanks, Editor; Teresa Ramsey, Editor, *Preventing Chronic Disease*. Disclosure: Caran Wilbanks and Teresa Ramsey have disclosed no relevant financial relationships.

**CME AUTHOR**

Charles P. Vega, MD, Health Sciences Clinical Professor; Residency Director, Department of Family Medicine, University of California, Irvine. Disclosure: Charles P. Vega, MD, has disclosed no relevant financial relationships.

**AUTHORS AND CREDENTIALS**

Disclosures: S. Bryn Austin, ScD; Jennifer L. Spadano-Gasbarro, PhD; Mary L. Greaney, PhD; Emily A. Blood, PhD; Anne T. Hunt, ScD; Tracy K. Richmond, MD, MPH; Monica L. Wang, MS; Solomon Mezgebu, MS; Stavroula K. Osganian, MD, ScD; Karen E. Peterson, ScD have disclosed no relevant financial relationships.

Affiliations: S. Bryn Austin, Emily A. Blood, Tracy K. Richmond, Stavroula K. Osganian, Boston Children’s Hospital, Boston, Massachusetts; Jennifer L. Spadano-Gasbarro, Mary L. Greaney, Monica L. Wang, Karen E. Peterson, Harvard School of Public Health, Boston, Massachusetts; Anne T. Hunt, Hunt Consulting Associates, Lyme, New Hampshire; Solomon Mezgebu, MS, Massachusetts Department of Public Health, Boston, Massachusetts.

## Introduction

Obesity in childhood and adolescence is a well-recognized public health concern. The percentage of children who are obese has doubled or even tripled in some countries over the past few decades (1). Disordered weight control behavior (DWCB), such as self-induced vomiting or abuse of laxatives or diet pills for weight control, and clinical and subclinical eating disorders are also recognized as international public health concerns (2). In the United States, the Centers for Disease Control and Prevention estimates that each month 6.0% of girls and 2.5% of boys vomit or abuse laxatives to control their weight (3). The myriad links between obesity, efforts to lose or control weight, and DWCB have been well described (4–6), and prevention researchers have begun to develop programs to prevent both obesity and DWCB in a single intervention.

In randomized controlled trials (RCTs), several behavioral interventions have demonstrated preventive effects on both obesity or obesity-related behaviors and DWCB in youth (7). For instance, New Moves, a 1-year, school-based exercise and nutrition intervention for high school girls, improved physical activity and dietary behaviors and reduced unhealthy weight control behaviors (8). Another program, Planet Health, an obesity prevention curriculum for middle schools, had preventive effects on both obesity and DWCB (9–11). Planet Health reduced the risk of developing DWCB in middle-school girls by half over a 2-year period, first in an efficacy trial (9) and then again in an effectiveness trial (10). Planet Health is both cost-effective and cost saving through its combined preventive effect on obesity and DWCB (12,13). Furthermore, the curriculum was found to be feasible and acceptable to school staff in an evaluation of diffusion of the program in urban middle schools (14). The curriculum is publicly available at low cost ($59; www.planet-health.org/). Since 2001, more than 10,000 copies of the curriculum have been sold and distributed in all 50 US states and in more than 20 countries (15).

The RCT study design has the benefit of evaluating the preventive program effects under ideal conditions where investigators exercise a great deal of control over implementation. These conditions maximize internal validity and strengthen RCT conclusions that observed effects are due to the intervention (16,17). Once preventive programs are shown to be beneficial in RCTs, first through efficacy trials and then through effectiveness trials, the next step is to move a program to scale (18,19). Scaling up programs on the order needed to achieve widespread benefit may require dissemination via collaborations with public sector and community nonprofit systems, such as schools or community centers, or the private sector, such as industry or private health insurers (20). Existing community systems needed for dissemination, however, may have many competing demands on staff time and resources and may lack financial or administrative support for staff to adhere to evidence-based protocols (18,21). Variability in implementation fidelity can lead to different results than those found with RCTs. Effective public health practice requires that programs be evaluated continuously throughout the process of translation from RCT to dissemination (18,21).

Researchers in cancer and obesity prevention, physical activity promotion, and numerous other health domains are increasingly focusing on evaluation of dissemination efforts (21–24). To date, however, no large-scale dissemination studies of evidence-based programs preventing DWCB have been reported. Therefore, our study was undertaken to evaluate the effect of Planet Health on DWCB when widely disseminated to middle schools. We hypothesized that higher fidelity of implementation would be associated with lower odds of DWCB after the intervention.

## Methods

In 2004, the Massachusetts Department of Public Health (MDPH) and Blue Cross Blue Shield of Massachusetts collaborated to disseminate the Planet Health curriculum via Healthy Choices (HC), a multicomponent intervention designed to prevent obesity in middle-school students (25,26). MDPH notified administrators from all of the state’s approximately 300 public middle schools that they could take part in HC. Forty-nine schools throughout the state received funding to implement HC in their schools over 3 years (fall 2005–spring 2008), and 45 of these schools participated in an evaluation led by the Harvard School of Public Health (HSPH) and Boston Children’s Hospital. The racial/ethnic composition of the HC sample was similar to that of students attending Massachusetts public schools as a whole, and the percentage eligible for free or reduced-priced school lunch in HC schools, 27.6%, was similar to the state average of 25.2% (27). MDPH provided schools with a letter that school principals could send home to parents requesting passive consent. The Institutional Review Board of HSPH approved the evaluation study with passive parental consent and adolescent assent at survey administration.

### Planet Health and the Healthy Choices program

The goals of HC were to prevent obesity in middle-school students through decreased television viewing and improved nutrition and physical activity. A central component of the program was the Planet Health curriculum, which was designed to meet academic curricular guidelines of the school system. The curriculum incorporates health messages on consuming more fruits and vegetables and less saturated and trans fat, being physically active, and reducing television viewing through 8 classroom lessons for each of the core academic areas (social sciences, language arts, math, and social studies), with an additional 30 microunits (ie, lessons lasting only 5 to 10 minutes) and 6 fitness checks in physical education (PE) classes (15). Additional details about Planet Health have been described previously (11). The curriculum does not explicitly discuss obesity or DWCB.

In addition to Planet Health, HC included before- and after-school programs, environmental and policy changes, school-wide promotions, and the School Health Index, a school self-assessment tool developed by the Centers for Disease Control and Prevention to help staff identify strengths and weaknesses in the school’s programs and policies (www.cdc.gov/healthyyouth/shi/). We encouraged schools to create partnerships with outside groups to help implement and sustain HC activities (26).

### Data sources

Anonymous, student self-report surveys assessing sociodemographics and various health behaviors were administered by school personnel to all students in grades 6 through 8 at baseline in the fall of 2005 and at Year 3 follow-up in the spring of 2008. All students attending school on the day of the survey were invited to participate, and only a nominal number opted out. Progress reports were completed by school staff in Year 2 in the spring of 2007, providing details on implementation of Planet Health and the other HC components and involvement of partners from outside the school in implementation activities. We used publicly available data from the National Center for Education Statistics to obtain socioeconomic information about each school (http://nces.ed.gov/datatools/index.asp?DataToolSectionID=4).

### Measures

#### Outcome variable: DWCB (individual-level)

The DWCB outcome was dichotomous; it was measured at the individual level and created by combining information from a 3-part item on the Year 3 student self-report survey, asking, “In the last 30 days, have you done any of the following to lose or maintain your weight?” The survey offered these response options: “Vomit or throw up on purpose after eating,” “Take laxatives,” “Take diet pills without a doctor’s permission.” We coded a report of 1 or more of these behaviors as affirmative for DWCB. Similarly worded items have been validated by the Centers for Disease Control and Prevention (28).

#### Primary predictor variables: Planet Health implementation (school-level)

We assessed implementation of the Planet Health curriculum at the school level with 8 variables created from information provided by school staff via the Year 2 progress report. The variables quantified the number of Planet Health lessons taught related to nutrition, television viewing, and physical activity goals; number of student lesson-exposures in each behavioral domain (eg, 100 students exposed to 2 nutrition lessons plus 50 students exposed to 3 nutrition lessons = 350 student lesson-exposures in the nutrition domain); number of PE microunits taught; and number of teachers trained in Planet Health.

#### Other implementation variables: HC components (school-level)

We assessed other HC components via the Year 2 progress reports. These variables quantified activities supporting behavioral goals through before- and afterschool programs, environmental and policy changes, and school-wide promotions for students and programs for staff; involvement of food service directors, PE teachers, administrators, students, and outside partners in HC activities; and level of teamwork among staff implementing HC. We encouraged schools to form a team that met regularly to implement HC, and we classified a school as meeting the definition of *team* if the HC coordinator for a school reported that staff working on HC met as a group, had a shared vision regarding HC, and had short-term and long-term goals.

#### Covariates (school-level)

All covariates included in models were school characteristics assessed at baseline. Covariates based on the student self-report survey were percentage of respondents in a school who were white, percentage who were female, percentage reporting DWCB, and number of respondents in grades 6 through 8. In addition, a covariate representing the percentage eligible for free or reduced-price lunch was based on data from the National Center for Education Statistics.

### Statistical analyses

We conducted multivariate logistic analyses with generalized estimating equations to examine the association of Planet Health implementation measures with the odds of students reporting DWCB at Year 3 follow-up. Note that because data were anonymous, models did not estimate change in individuals’ DWCB from baseline to follow-up and were based on 2 sequential cross-sectional samples with substantially but not entirely overlapping samples. First, each Planet Health implementation variable and other HC implementation variables were examined individually in models controlling for school-level baseline DWCB prevalence and other covariates described above. Next, a saturated model was constructed including all variables, and then through a process of backward elimination, any HC implementation variables that did not reach *P* < .05 were dropped from the model while all Planet Health variables and baseline school-level covariates were retained. Finally, after the process of backward elimination, we identified a fully adjusted model that included all Planet Health and HC implementation variables that remained significant (*P* < .05), controlling for baseline school-level covariates. We used generalized estimating equation methods to account for school clustering.

At baseline, 20,710 students responded to the anonymous student self-report survey, but, because of missing information, school-level covariates were based on 20,363 surveys (98.3% of total). Students contributing data to baseline school-level covariates compared with those who did not contribute data did not differ by sex, race/ethnicity, grade, or DWCB (all *P* > .05). At Year 3 follow-up, 17,567 students responded to the self-report survey. The DWCB outcome variable is based on data from 16,369 students who responded to this item at follow-up (93.2% of total).

## Results

Participating middle schools varied widely on school characteristics and prevalence of students reporting DWCB (Table 1). The percentage of students reporting DWCB ranged from 0.9% to 12.0% at baseline and from 0% to 14.0% at Year 3 follow-up, but the mean percentage reporting DWCB across the schools did not differ from baseline to follow-up (4.2% vs 4.6%; *P* > .05). The distribution of variables representing Planet Health and other HC intervention components as measured at Year 2 had large ranges across schools in implementation for every component (Table 2). The HC team met our definition of team in 24 of the schools, and 2 schools had programs addressing television goals with school staff.

**Table 1:** Distribution of School Sociodemographic Characteristics and Disordered Weight-Control Behavior^a^ at Baseline and Year 3 Follow-up in Massachusetts Middle Schools (N = 45) Participating in the Healthy Choices Program, 2005–2008

Characteristics of Schools	Mean (SD)	Range
% White students at baseline	70.3 (23.1)	4.3–95.4
% Female students at baseline	50.6 (4.4)	34.4–60.3
% Students eligible for free or reduced-price lunch at baseline	27.6 (20.0)	2.4–85.6
No. of respondents in grades 6–8	484 (303)	16–1,277
% Students with DWCB^a^ at baseline	4.2 (2.5)	0.9–12.0
% Students with DWCB^a^ at follow-up	4.6 (2.5)	0–14.0

a Disordered weight-control behavior (DWCB) defined as report of any vomiting or use of laxatives or diet pills for weight control in past month.

**Table 2:** Distribution of Variables Representing Planet Health and Other Healthy Choices (HC) Intervention Components at Year 2 in Massachusetts Middle Schools (N = 45) Participating in the HC Program, 2005–2008

Variables	Mean (SD)	Range
No. of Planet Health lessons taught on nutrition	11.09 (12.63)	0–49
No. of student lesson-exposures to Planet Health lessons on nutrition	295.16 (389.37)	0–1,797
No. of Planet Health lessons taught on reducing television viewing	2.62 (3.77)	0–15
No. of student lesson-exposures to Planet Health lessons on reducing television viewing	66.69 (98.90)	0–380
No. of Planet Health lessons taught on physical activity and fitness	7.11 (9.79)	0–43
No. of student lesson-exposures to Planet Health lessons on physical activity and fitness	384.62 (764.71)	0–4,749
No. of Planet Health PE microunits^a^ taught	4.22 (5.72)	0–22
No. of teachers teaching Planet Health lessons	5.82 (6.18)	0–24
No. of partners from outside school involved in HC activities	0.38 (0.78)	0–3

a Lessons lasting only 5 to 10 minutes.

In the fully adjusted model, for each additional 100 student lesson-exposures to Planet Health lessons on reducing television viewing, a student’s odds of DWCB at follow-up were reduced by 20% (Table 3). In addition, we found that a greater number of partners from outside the school involved in HC activities (24% reduced odds of DWCB for each additional partner), the presence of a program addressing television viewing reduction goals with staff (62% reduced odds of DWCB), and having program staff who met the study definition of a team (24% reduced odds of DWCB) all had protective effects on odds of DWCB at follow-up. We found small but significant positive associations for greater number of Planet Health lessons taught on television-viewing reduction, greater number of student lesson-exposures to Planet Health lessons on physical activity, and greater number of teachers teaching Planet Health lessons (Table 3).

**Table 3:** Multivariate Models Estimating Odds Ratios of Reporting Disordered Weight-Control Behavior^a^ at Follow-up Associated With Planet Health and Other Healthy Choices (HC) Intervention Components in 45 Massachusetts Middle Schools, 2005–2008 (N = 17,567 Students)^b^

Variable	Partially Adjusted Models^c^, OR (95% CI)	*P* Value	Fully Adjusted Model^d^, OR (95% CI)	*P* Value
No. of Planet Health lessons taught on nutrition	1.01 (0.99–1.02)	.42	**—**	**—**
No. of student lesson-exposures to Planet Health lessons on nutrition (per 100 lesson-exposures)	1.01 (0.97–1.04)	.67	**—**	**—**
No. of Planet Health lessons taught on reducing television viewing	1.00 (0.97–1.03)	.96	1.04 (1.02–1.07)	<.001
No. of student lesson-exposures to Planet Health lessons on reducing television viewing (per 100 lesson-exposures)	0.94 (0.82–1.07)	.36	0.80 (0.74–0.85)	<.001
No. of Planet Health lessons taught on physical activity and fitness	1.00 (0.99–1.01)	.93	**—**	**—**
No. of student lesson-exposures to Planet Health lessons on physical activity and fitness (per 100 lesson-exposures)	1.01 (1.00–1.02)	.003	1.01 (1.00–1.01)	.008
No. of Planet Health physical education microunits^e^ taught	1.00 (0.99–1.01)	.82	**—**	**—**
No. of teachers teaching Planet Health lessons	0.99 (0.97–1.02)	.47	1.02 (1.01–1.03)	.002
No. of partners from outside school involved in HC activities	0.73 (0.62–0.86)	<.001	0.76 (0.68–0.84)	<.001
Presence of programs addressing television viewing reduction goals with school staff	0.54 (0.40–0.73)	<.001	0.38 (0.28–0.53)	<.001
HC team meets definition of *team*	0.77 (0.68–0.95)	.02	0.76 (0.66–0.86)	<.001

Abbreviations: OR, odds ratio; CI, confidence interval; **—**, not applicable.

a Disordered weight-control behavior defined as report of any vomiting or use of laxatives or diet pills for weight control in past month.

b Both models use individual level as unit of analysis and generalized estimating equation methods to account for school clustering.

c Partially adjusted models adjust for school sociodemographic characteristics listed in Table 1 and disordered weight-control behavior at baseline.

d Fully adjusted model adjusts for all variables in the table that remained significant at *P* < .05 after process of backward elimination, school sociodemographic characteristics, and disordered weight-control behavior at baseline.

e Lessons lasting only 5 to 10 minutes.

## Discussion

Successful translation of preventive programs from the RCT phase of research to large-scale public health initiatives requires continued evaluation throughout the dissemination process (18,21). Such initiatives often also require cross-sector collaboration, including some combination of public, nonprofit, and private sector partners (20). Our study builds on prior RCT studies (9–11) that established the protective effect of the Planet Health obesity-prevention curriculum on DWCB in middle schools by evaluating its implementation as part of a large-scale dissemination program led by a state public health department in collaboration with a major private health insurer in the state. This study appears to be both the first large-scale dissemination evaluation of the effects of an obesity prevention program on DWCB in youth and the first partnership between public and private sectors implementing an evidence-based DWCB prevention program statewide in the United States.

Findings indicated that when Planet Health was widely disseminated throughout Massachusetts as part of the HC program, a greater number of student lesson-exposures to Planet Health lessons on reducing television viewing was associated with reduced odds of student DWCB at follow-up. Students in schools offering programs for staff on reducing television viewing had reduced odds of DWCB at follow-up compared with students in other schools. It is possible that the staff programs may have enhanced the education that students received on why and how to reduce their own television viewing. The Planet Health efficacy trial found decreased television viewing was the strongest mediator of intervention effects on obesity (11). In our study, other factors protective against DWCB at follow-up were having a greater number of partners from outside the school involved with HC activities and having a high level of teamwork among staff working on HC.

Of concern is that we found positive associations for some Planet Health components, which is counter to study hypotheses; however, this is not surprising, given the challenges that schools face in implementing and sustaining complex, long-term health-promotion programs (16,21). A larger number of teachers teaching Planet Health lessons and a larger number of student lesson-exposures to the physical activity and fitness topics were both associated with small but significantly increased odds of DWCB at follow-up. The reasons underlying these associations are unclear, but it is possible that in schools where many teachers were teaching Planet Health, it was difficult to make sure all received training. In a separate process evaluation of HC, school staff sometimes found it difficult to ensure that teachers received training in the Planet Health curriculum even though training was offered both in person and online (ML Greaney, CK Hardwick, JL Spadano-Gasbarro, S Mezgebu, CM Horan, S Schlotterbeck, et al, unpublished data). In addition, prior research has found that many school teachers, like adults in general, hold stigmatizing attitudes toward obesity, and physical education and health teachers in particular are themselves at elevated risk of eating disorder symptoms and behaviors compared with other teachers (29,30). These findings suggest that school staff involved in implementing Planet Health and other obesity and DWCB prevention programs likely need training not only in how to implement the programs but also in how to recognize and address their own possible weight-biased attitudes and unhealthy beliefs and behaviors related to food and weight (29–31).

A greater number of Planet Health lessons taught on reducing television viewing also had a positive association with DWCB, but only after simultaneously controlling for number of student lesson-exposures to the television lessons, which, in contrast, was protective. Reasons for this seemingly paradoxical finding are unclear. Because these 2 variables were highly correlated and may be confounded, data from our study did not allow full disentanglement of their effects. One alternative interpretation is that if the number of television lesson-exposures is held constant, then increasing the number of Planet Health television lessons exposes students to a more diverse set of messages from teachers. In this light, it is possible that increasing lesson-exposures is protective, but only when done in a way that focuses the message and exposes the most children to the same narrow set of effective messages.

This study has several limitations. Survey data from students were anonymous and therefore did not allow estimation of change in individuals. Students attending the schools at baseline were overlapping but not identical to those at follow-up because of normal fluctuation resulting from graduation and changing residences. We did not use a randomized-controlled design; however, because Planet Health has already been shown to be protective against obesity and DWCB in efficacy and effectiveness RCTs (9–11), MDPH held that it would not be appropriate to withhold a proven intervention through randomization of schools. More frequent assessments to monitor incremental behavior changes were not possible. Information was not available on idiosyncratic implementation styles of individual teachers; for instance, some teachers who themselves hold unhealthy beliefs about food, weight, and weight control may have conveyed these beliefs to students while teaching Planet Health lessons (29,30). We did not assess potentially important symptoms, such as preoccupation with thinness, body dissatisfaction, fasting, and some symptoms that especially involve males, such as use of anabolic steroids.

Findings from our study provide modest evidence that Planet Health can help prevent DWCB when widely disseminated to middle schools by a cross-sector (ie, public-private) partnership as part of a multicomponent obesity prevention program. Messages related to reducing television viewing for both students and staff seem to be especially important. In addition, findings underscore the need to ensure adequate training for dissemination with regard to both implementation and staff biases. Further study of Planet Health and HC is needed to identify why some activities appear to be protective when implemented under real-world conditions while others are not and to identify what level and types of teacher training are required to achieve protective effects. With the increase in the number of programs to improve weight-related behaviors and prevent obesity and DWCB currently in efficacy trials, researchers need to turn next to effectiveness trials, and finally to dissemination studies, likely through cross-sector partnerships. Through these next steps, we can ensure that all youth benefit from evidence-based preventive programs.

## Figures and Tables

**Table:** 

**1. The activity supported the learning objectives.**				
**Strongly Disagree**				**Strongly Agree**
1	2	3	4	5
**2. The material was organized clearly for learning to occur.**				
**Strongly Disagree**				**Strongly Agree**
1	2	3	4	5
**3. The content learned from this activity will impact my practice.**				
**Strongly Disagree**				**Strongly Agree**
1	2	3	4	5
**4. The activity was presented objectively and free of commercial bias.**				
**Strongly Disagree**				**Strongly Agree**
1	2	3	4	5

## Post-Test Information

To obtain credit, you should first read the journal article. After reading the article, you should be able to answer the following, related, multiple-choice questions. To complete the questions (with a minimum 70% passing score) and earn continuing medical education (CME) credit, please go to http://www.medscape.org/journal/pcd. Credit cannot be obtained for tests completed on paper, although you may use the worksheet below to keep a record of your answers. You must be a registered user on Medscape.org. If you are not registered on Medscape.org, please click on the "Register" link on the right hand side of the website to register. Only one answer is correct for each question. Once you successfully answer all post-test questions you will be able to view and/or print your certificate. For questions regarding the content of this activity, contact the accredited provider, CME@medscape.net. For technical assistance, contact CME@webmd.net. American Medical Association's Physician's Recognition Award (AMA PRA) credits are accepted in the US as evidence of participation in CME activities. For further information on this award, please refer to http://www.ama-assn.org/ama/pub/category/2922.html. The AMA has determined that physicians not licensed in the US who participate in this CME activity are eligible for **AMA PRA Category 1 Credits™**. Through agreements that the AMA has made with agencies in some countries, AMA PRA credit may be acceptable as evidence of participation in CME activities. If you are not licensed in the US, please complete the questions online, print the AMA PRA CME credit certificate and present it to your national medical association for review.

## Post-Test Questions

### Article Title: Effect of the Planet Health Intervention on Eating Disorder Symptoms in Massachusetts Middle Schools, 2005—2008

**CME Questions**

1. You advise a school district regarding the health of its students. The district is planning to implement the Planet Health curriculum. Which of the following statements regarding this program is most accurate?
  - It has been demonstrated to reduce obesity but not disordered weight control behaviors (DWCB)
  - It has been demonstrated to reduce DWCB but not obesity
  - The program has only been used in suburban and affluent schools
  - The curriculum is available for less than $60
2. What happened to the prevalence of DWCB across the schools as a whole in the current study during the 3-year intervention period?
  - It fell by more than 60%
  - It fell by approximately 30%
  - It remained largely unchanged
  - It increased by approximately 30%
3. Which of the following interventions of the Planet Health program was most effective in reducing rates of DWCB in the current study?
  - Exercise instruction
  - Advice of fruit and vegetable consumption
  - Lessons on reducing television viewing
  - Advice on the consumption of fats
4. Which of the following statements regarding subgroup analysis of the current study is pertinent for this patient?
  - Female sex among students
  - More partners outside of school participating in the Healthy Choices program
  - Higher number of physical education sessions
  - More teachers providing the Planet Health curriculum

**Evaluation**
